# Oral glucose tolerance testing at 1 h and 2 h: relationship with glucose and cardiometabolic parameters and agreement for pre-diabetes diagnosis in patients with morbid obesity

**DOI:** 10.1186/s13098-022-00865-2

**Published:** 2022-07-06

**Authors:** Vanessa Guerreiro, Isabel Maia, João Sérgio Neves, Daniela Salazar, Maria João Ferreira, Fernando Mendonça, Maria Manuel Silva, Marta Borges-Canha, Sara Viana, Cláudia Costa, Jorge Pedro, Ana Varela, Eva Lau, Paula Freitas, Davide Carvalho, AMTCO Group

**Affiliations:** 1 Departamento de Endocrinologia, Diabetes E Metabolismo, Centro Hospitalar Universitário São João, Alameda Prof. Hernâni Monteiro, 4200-319 Porto, Portugal; 2 Faculdade de Medicina, Universidade Do Porto, Porto, Portugal; 3 Instituto de Investigação E Inovação Em Saúde, Universidade Do Porto, Porto, Portugal; 4 EPIUnit, Instituto de Saúde Pública, Universidade Do Porto, Porto, Portugal; 5 Serviço de Medicina Interna, Unidade Local de Saúde Do Norte Alentejano, Portalegre, Portugal; 6 Serviço de Endocrinologia, Instituto Português de Oncologia, Porto, Portugal; 7 Consulta de Avaliação Multidisciplinar Do Tratamento Cirúrgico da Obesidade Do Centro Hospitalar Universitário São João, Porto, Portugal

**Keywords:** Oral glucose tolerance testing, Prediabetes *mellitus*, Obesity

## Abstract

**Background:**

One hour plasma glucose concentration (1hPG) during an oral glucose tolerance test (OGTT) may be an alternative to 2-h plasma glucose concentration (2hPG) in the identification of individuals at increased risk of hyperglycaemia, although its role is not fully understood.

**Aim:**

We aim to investigate the relationship of these measures with other glucose parameters, as well as their relationship with cardiometabolic risk markers and the level of agreement for prediabetes *mellitus* diagnosis, in a sample of patients with morbid obesity.

**Methods:**

We retrospectively evaluated 656 patients with morbid obesity without diagnosed diabetes. To define prediabetes with 2hPG, 2022 American Diabetes Association guidelines criteria were used, while for 1hPG, glucose ≥ 155 mg/dL was considered. Cohen’s Kappa coefficient was used to assess the agreement between both measures of prediabetes *mellitus* diagnosis.

**Results:**

A Cohen’s Kappa coefficient of 0.405 (p < 0.001) was obtained. The 1hPG were positively correlated with homeostatic model assessment for insulin resistance (HOMA-IR) (*ρ* = 0.281, *p* < 0.001), fasting plasma glucose (FPG) (*ρ* = 0.581, *p* < 0.001), glycated haemoglobin (Hb1AC) (*ρ* = 0.347, *p* < 0.001) and were negatively correlated with homeostatic model assessment for cell-β function (HOMA-β) (*ρ* = −0.092, *p* = 0.018). 2hPG were also correlated with the same parameters, except for HOMA-β.

**Conclusion:**

A fair agreement between 1 and 2hPG was verified. 1hPG criteria may be a useful indicator of β-cell dysfunction and insulin resistance in patients with morbid obesity without diabetes diagnosis.

## Introduction

Over the last decades, the prevalence of obesity has increased worldwide, whose consequences are well-recognised and include several comorbidities, such as type 2 diabetes *mellitus* (T2DM) [1–3]. T2DM represents a key determinant of cardiovascular morbidity and mortality in the adult population [4, 5], as such, early detection of individuals at high risk of developing this disease (individuals with prediabetes *mellitus*) is paramount for the prevention not only of T2DM but also of cardiovascular disease (CVD).

Fasting plasma glucose (FPG), 2-h plasma glucose concentration (2hPG) during an oral glucose tolerance test (OGTT), and glycated haemoglobin (HbA1c) are all currently used to diagnose prediabetes and diabetes—however, the agreement between these three methods is imperfect [6]. On the other hand, the 2hPG has been shown to diagnose more people with prediabetes and diabetes [7].

It has been reported that a relatively large number of people develop diabetes *mellitus* without being diagnosed the preceding stage of prediabetes [8]. Therefore, the use of the current definition of normal glucose tolerance (NGT) based on the FPG, 2hPG, or HbA1c level could lead to not diagnosing many people who are at increased risk of developing T2DM. Thus, alternative ways to identify T2DM have been studied.

Recent reports have identified that 1-h plasma glucose concentration (1hPG) ≥ 155 mg/dL during the OGTT is associated with future risk for T2DM [9–12] and also identifies subjects with a worse cardiometabolic profile [13–15]. The 1hPG is well accepted and used to screen pregnant women for gestational diabetes [16]. Logistically, 1hPG is less resource-intensive than 2hPG. If its value is equivalent to the 2hPG in identifying risk, then the 1hPG would be a useful measure as it is a less-burdensome test.

Recently, Paddock et al*.* compared the 1hPG and 2hPG in predicting diabetic retinopathy and showed that both were similar in identifying this pathology, recommending that 1hPG should be adopted as an alternative method to identify those at risk from this complication [17]. However, few studies have been conducted in exploring the agreement of 1hPG and 2hPG [18], especially in patients with morbid obesity, as well as its relationship with cardiometabolic and glycaemic parameters. Therefore, this study had as primary objective to evaluate the 1hPG and 2hPG agreement in pre-T2DM diagnosis and as secondary objective its relationship with parameters of glucose metabolism and cardiometabolic risk. We also investigated whether patients with normal glucose homeostasis by OGTT with 1hPG ≥ 155 mg/dL have a worse cardiometabolic profile than those with 1hPG < 155 mg/dL.

## Material and methods

### Study design and participants

We carried out a retrospective observational study including all patients with morbid obesity evaluated to bariatric surgery (BS)—Roux-en-Y gastric bypass (RYGB), a laparoscopic adjustable gastric band (LAGB), or a laparoscopic sleeve gastrectomy (LSG)—between January 2010 and July 2018 in São João University Hospital Centre. We excluded patients with T2DM criteria and without preoperative assessment of fasting blood glucose levels, 1hPG or 2hPG. A sample of 656 patients who were referred for BS during this period was included in this study.

All study participants either had a body mass index (BMI) > 40 kg/m^2^ or an obesity-related comorbidity and BMI > 35 kg/m^2^. Information on preoperative clinical visits was collected from the patient’s clinical file.

All procedures performed in the study were in accordance with the ethical standards of the national guidelines.

### Clinical parameters and other characteristics

The following preoperative parameters were collected: age, sex, and anthropometric measurements [including height, weight to calculate BMI, and waist (WC) and hip circumferences (HC)] all of which were measured using standardised techniques. Systolic (SBP) and diastolic blood pressure (DBP) were also retrieved, being measured using a mercury sphygmomanometer. The 2022 American Diabetes Association guidelines criteria and glucose ≥ 155 mg/dL were used to define pre-T2DM, for 2hPG and 1hPG, respectively. Data on smoking habits was also retrieved.

### Routine laboratory tests

In addition to clinical parameters, data on HbA1c, serum insulin, creatinine, transaminases, as well on lipid profile [triglycerides, total cholesterol, high-density lipoprotein cholesterol (HDL-C) and low-density lipoprotein cholesterol (LDL-C)], were also taken from the hospital clinical files. Values of HOMA-IR [(fasting serum glucose, mg/dL) × (fasting serum insulin, µU/mL)/405] were used as a measure of insulin resistance (IR) [19] and values of HOMA-β [20 × fasting serum insulin, µU/mL)]/[(fasting serum glucose, mmol/L)–3.5)] were used as a measure of β-cell function [19]. The OGTT was performed using a standardised protocol and blood samples were drawn at both in fasting and after 1 h and 2 h of 75-g OGTT regarding glucose and C-peptide, after overnight fasting [18]. As part of the OGTT, based on insulin and glucose levels at the beginning and after 30 min, the insulinogenic index, as the ratio between the difference between insulin at 30 min and the begging and the difference between glucose at 30 min and at the beginning was calculated to evaluate the insulin response [20].

### Statistical analysis

Continuous variables were described as mean and standard deviation (SD), or as median and interquartile range (IQR), and were compared using Student’s T-test or the Mann–Whitney test, as appropriate. Categorical variables were summarised as counts and proportions. The Cohen’s Kappa coefficient was computed to evaluate the agreement between the two measures of pre-T2DM diagnosis—1hPG and 2hPG (primary endpoint). For the secondary objective, correlations between continuous variables (the relationship of 1hPG and 2hPG with parameters of glucose metabolism and cardio-metabolic risk) were carried out using the Pearson (*r*) correlation coefficient or the Spearman (ρ) correlation coefficient, as appropriate. Statistical analysis was performed using SPSS Statistics 25.0 (IBM Corp, Armonk, New York). A significance level of 5% was considered.

## Results

The studied sample was mainly composed of women (86.9%) and had a mean ± SD age of 40.3 ± 10.1 years (Table 1). The mean preoperative BMI was 43.6 ± 5.5 kg/m^2^, the WC 121.6 ± 13.0 cm and HC 132.3 ± 10.5 cm. The mean fasting glucose was 93.1 ± 10. 8 mg/dL, 1hPG 154.7 ± 37.7 mg/dL and 2hPG 123.2 ± 27.9 mg/dL (Table 1).

**Table 1:** Baseline characteristics of the studied sample (n = 656)

	n	
Sex (n (%))	656	
Male		86 (13.1)
Female		570 (86.9)
Age [years, mean (SD)]	655	40.3 (10.1)
BMI [kg/m^2^, mean (SD)]	655	43.6 (5.5)
Waist circumference [cm, mean (SD)]	497	121.6 (13.0)
Hip circumference [cm, mean (SD)]	470	132.3 (10.5)
SBP [mmHg, mean (SD)]	521	134.9 (17.8)
DBP [mmHg, mean (SD)]	520	83.8 (10.9)
Total cholesterol (37)	645	195.9 (37.5)
HDL-C (37)	641	49.5 (11.0)
LDL-C (37)	633	121.6 (31.4)
Triglycerides [mg/dL, median (IQR)]	649	112.0 (68.0)
hsCRP [mg/dL, median (IQR)]	469	8.9 (10.2)
Creatinine [mg/dL, median (IQR)]	640	0.6 (0.2)
AST [U/L, median (IQR)]	611	22.0 (9.0)
ALT [U/L, median (IQR)]	646	22.0 [16]
HbA1c [%, mean (SD)]	656	5.4 (0.4)
Glucose OGTT (fasting) [37]	656	93.1 (10.8)
Glucose OGTT [30 min] (37)	655	145.9 (26.4)
Smoking habits	589	
No		512 (78.0)
Yes		77 (11.7)

*ALT* Alanine aminotransferase, *AST* Aspartate aminotransferase, *BMI* body mass index, *DBP* diastolic blood pressure, *HbA1c* glycated haemoglobin, *HDL*-*c* high-density lipoprotein cholesterol, *hsCRP* high sensitivity C-reactive protein, *LDL*-*c* low-density lipoprotein cholesterol, *OGTT* oral glucose tolerance test, *SBP* systolic blood pressure, *SD* standard deviation

Using the Cohen’s Kappa coefficient, the agreement between the 1hPG and 2hPG in diagnosing pre-T2DM was explored. In our sample of 656 patients, 153 are classified as pre-T2DM by both tests, 163 patients with normoglycemia at 2hPG are classified as having pre-T2DM according to 1hPG vs 29 with normoglycemia in 1hPG and with pre-T2DM criteria by 2hPG. The value of the Cohen’s Kappa coefficient was 0.405 (*p* < 0.001), corresponding to a fair agreement between 1 and 2hPG (Table 2).

**Table 2:** Analysis of agreement between 1 and 2hPG

	2hPG		Total
	< 140 mg/dL	≥ 40 mg/dL	
1hPG			
< 155 mg/dL	311	29	340
≥ 55 mg/dL	163	153	316
Total	474	182	656

Kappa coefficient = 0.405 (*p* < 0.001)

*1hPG* 1-h plasma glucose concentration, *2hPG* 2-h plasma glucose concentration

The values of 1hPG showed to be positively correlated with HOMA-IR (*ρ* = 0.281, *p* < 0.001), fasting glucose (*ρ* = 0.581, *p* < 0.001) and HbA1c (*ρ* = 0.347, *p* < 0.001), and negatively correlated with HOMA-β (*ρ* = −0.092, *p* = 0.018). Values of 2hPG were also correlated with the same variables, except HOMA-β (*ρ* = 0.029, *p* = 0.462). The insulinogenic index showed to be negatively correlated with both 1hPG (*ρ* = −0.391, *p* < 0.001) and 2hPG (*ρ* = −0.259, *p* < 0.001) (Table 3).

**Table 3:** Correlations between 1 and 2hPG and glucose and cardiometabolic parameter

Parameter	Fasting glucose		1hPG		2hPG	
	Correlation coefficient*	*p*-value	Correlation coefficient*	*p*-value	Correlation coefficient*	*p*-value
Fasting glucose (mg/dL)	−	–	0.581	< 0.001	0.359	< 0.001
HOMA-β	−0.310^†^	< 0.001	−0.092^†^	0.018	0.029^†^	0.462
HOMA-IR	0.312^†^	< 0.001	0.281^†^	< 0.001	0.254^†^	< 0.001
HbA1c (%)	0.354	< 0.001	0.347	< 0.001	0.221	< 0.001
Peptide C (ng/mL)	0.293	< 0.001	0.315	< 0.001	0.238	< 0.001
Triglyceride (mg/dL)	0.093^†^	0.018	0.247^†^	< 0.001	0.226^†^	< 0.001
HDL-cholesterol (mg/dL)	−0.093	0.018	−0.099	0.012	−0.041	0.300
LDL-cholesterol (mg/dL)	0.047	0.234	0.043	0.286	0.043	0.283
Total-cholesterol (mg/dL)	0.039	0.326	0.069	0.078	0.078	0.047
hsCRP (mg/dL)	−0.037^†^	0.430	0.061^†^	0.186	0.192^†^	< 0.001
Creatinine (mg/dL)	−0.027^†^	0.502	−0.018^†^	0.652	−0.061^†^	0.126
Insulinogenic index	−0.255^†^	< 0.001	−0.391^†^	< 0.001	−0.259^†^	< 0.001
SBP (mmHg)	0.147	< 0.001	0.138	0.002	0.106	0.015
DBP (mmHg)	0.110	0.012	0.077	0.080	0.038	0.386

*1hPG* 1-h plasma glucose concentration, *2hPG* 2-h plasma glucose concentration, *DBP* Diastolic blood pressure, *HbA1c* glycated haemoglobin, *HDL*-*c* high-density lipoprotein cholesterol, *HOMA*-*β* homeostatic model assessment for cell-β function, *HOMA*-*IR* homeostatic model assessment for insulin resistance, *hsCRP* high sensitivity C-reactive protein, *LDL*-*c* low-density lipoprotein cholesterol, *PG* post-load glucose, *SBP* Systolic blood pressure

*Pearson correlation coefficient, or otherwise specified

^†^Spearman correlation coefficient

Patients with 1hPG ≥ 155 mg/dL had higher levels of fasting glucose (98.0 mg/dL vs 88.6 mg/dL; *p* < 0.001), HbA1c (5.6%—38 mmol/mol vs 5.3%—34 mmol/mol, *p* < 0.001), HOMA-IR (4.3 vs 3.3, *p* < 0.001) and of C-Peptide (3.9 ng/mL vs 3.3 ng/mL, *p* < 0.001), and lower HOMA-β (212.1 vs 251.5, *p* = 0.003) (Table 3). HOMA-β is not significant different in patients with 2hPG ≥ 140 mg/dL vs  < 140 mg/dL (222.3 vs 204.3, *p* = 0.402) (Table 4).

**Table 4:** Glucose profile between patients with 1hPG < 155vs  ≥ 155 mg/dL and with 2hPG < 140 mg/dL*v* ≥ 140 mg/dL

Glucose 1 h (mg/dL)	1hPG				2hPG			
	n	< 155	≥ 155	p-value	n	< 140	≥ 140	p-value
1hPG [mg/dL, mean (SD)]	–	–	–	–	656	143.4 (33.8)	184.2 (30.8)	< 0.001
2hPG [mg/dL, mean (SD)]	656	109.7 (21.4)	137.7 (26.9)	< 0.001	**–**	**–**	**–**	**–**
Fasting glucose [mg/dL, mean (SD)]	656	88.6 (9.5)	98.0 (10.0)	< 0.001	656	91.3 (10.3)	97.8 (10.8)	< 0.001
HbA1c [%, mean (SD)]	656	5.3 (0.4)	5.6 (0.4)	< 0.001	656	5.4 (0.4)	5.6 (0.4)	< 0.001
Peptide C [ng/mL, mean (SD)]	629	3.3 (1.1)	3.9 (1.3)	< 0.001	629	3.5 (1.2)	4.0 (1.3)	< 0.001
Homa-IR [median (IQR)]	654	3.3 (2.4)	4.3 (3.4)	< 0.001	654	3.4 (2.7)	4.6 (3.1)	< 0.001
HOMA-β [median (IQR)]	654	251.5 (216.0)	212.1 (205.0)	0.003	654	226.5 (204.3)	237.0 (222.3)	0.402
Insulinogenic index (median (IQR))	643	1.66 (1.54)	1.02 (0.89)	< 0.001	643	1.42 (1.43)	1.02 (0.88)	< 0.001

*1hPG* 1-h plasma glucose concentration, *2hPG* 2-h plasma glucose concentration, *HbA1c* glycated haemoglobin, *HOMA*-*β* homeostatic model assessment for cell-β function, *HOMA*-*IR* homeostatic model assessment for insulin resistance, *IQR* Interquartile range, *PG* post-load glucose, *SD* standard deviation

In a sensitivity analysis, after excluding patients with pre-T2DM criteria according to fasting glucose, HbA1c and 2hPG, patients with 1hPG ≥ 155 mg/dL had higher triglycerides levels (125.0 vs 99.0). No significant differences were observed regarding HDL, LDL, total cholesterol, high sensitivity C-reactive protein (hsCRP), creatinine, HOMA-IR or HOMA-β (Table 5).

**Table 5:** Comparison between 1 h post-load glucose < 155 vs  ≥ 155 mg/dL in subjects with normal glucose tolerance (n = 295)

		Glucose 1 h (mg/dL)		*p*-value
		< 155 mg/dL	≥ 55 mg/dL	
Triglycerides [mg/dL, median (IQR)]	293	99.0 (64)	125.0 (92)	0.011
HDL-cholesterol (37)	292	51.1 (12.1)	50.6 (11.9)	0.779
LDL-cholesterol (37)	289	117.1 (31.5)	121.0 (3.9)	0.373
Total-cholesterol (37)	294	190.9 (37.1)	198.6 (39.7)	0.138
hsCRP [mg/dL, median (IQR)]	223	8.3 (10.2)	7.6 (9.8)	0.464
Creatinine [mg/dL, median (IQR)]	289	0.6 (0.2)	0.6 (0.2)	0.488
HOMA-IR [median (IQR)]	295	3.1 (2.3)	3.5 (3.4)	0.103
HOMA-β [median (IQR)]	295	263.6 (217.1)	233.1 (220.7)	0.549
Insulinogenic index [median (IQR)]	288	1.78 (1.75)	1.10 (1.29)	< 0.001

*HDL* high-density lipoprotein cholesterol, *HOMA*-*β* homeostatic model assessment for cell-β function, *HOMA*-*IR* homeostatic model assessment for insulin resistance, *hsCRP* high sensitivity C-reactive protein, *LDL* low-density lipoprotein, *IQR* Interquartile range, *SD* standard deviation

## Discussion

In this study, a fair agreement between 1 and 2hPG was verified. Indeed, agreement between the two methods varied between studies [6, 21, 22]. However overall it appears that the more altered 2hPG is, the more patients will be identified as having prediabetes with 1hPG [21], that is, some agreement between the methods have been demonstrated, as in our study, although imperfect. The existing definitions of pre-T2DM have different sensitivities and specificities for the identification of patients, even though the populations identified are somewhat overlapping [6].

According to the literature, the proportion of patients who progress to diabetes seems to differ between the definitions of pre-T2DM and with the possibility of an HbA1c of 6.0–6.4% (42–46 mmol/mol) identifying individuals at lower risk than the other criteria, which are currently in use [23]. 1hPG seems to identify patients with dysglycemia earlier [9, 10, 12, 24–26] and it has also been previously reported (in patients with overweight) that fasting glucose and the 2hPG were weak predictors for the risk of future T2DM compared to the 1hPG [22].

Regarding our secondary objectives, we observed that 1hPG values were negatively correlated with β-cell function and insulinogenic index and were positively correlated with IR, fasting glucose and HbA1c. Similar results were obtained for 2hPG, except for HOMA-β. Looking for the correlation coefficients, overall, the correlations seemed to be stronger for 1hPG than 2hPG. Bergman et al*.*[15] showed that the 1hPG is associated with an increased risk of mortality, even when the 2hPG is lower than 140 mg/dl. Furthermore, they demonstrated that individuals at risk of developing diabetes can be earlier identified using the 1hPG cut-off point of 155 mg/dL. Also, a retrospective cohort study carried out in China reported that 1hPG has better sensitivity and specificity than fasting glucose or 2hPG in predicting T2DM in subjects with normal glucose homeostasis [27]. Other studies [14, 28] have also shown that a 1hPG cut-off of equal to or higher than 155 mg/dL correlates with impaired β-cell function, lower insulin sensitivity, and a greater risk of developing T2DM and CVD. We also observed that 1hPG ≥ 155 mg/dL is associated with high triglyceride levels—even in patients with normal glucose homeostasis by OGTT.

The insulinogenic index on the OGTT can be used as an index of early phase insulin secretion [29, 30]. It was demonstrated that early insulin secretion plays a crucial role in maintaining normal glucose homeostasis [31], and patients with a low insulinogenic index could be more likely to developed of diabetes. In our study, the inverse correlation between the insulinogenic index and 1hPG was higher than between insulinogenic index and 2hPG, which could in some extent to be in favor of a greater potential of 1hPG in detecting early changes in glucose metabolism. Individuals with NGT with a high 1hPG could have reduced β-cell glucose sensitivity, but with a residual β-cell mass and preserved second phase insulin secretion, which results in maintaining the NGT. The subsequent loss of the second phase of insulin secretion could lead to impaired glucose tolerance, and finally to overt T2DM [6].

According to our findings, 1hPG were significantly and positively correlated with triglycerides, and negatively correlated with HDL-C. This is in accordance with the observation that dyslipidaemia in T2DM patients is characterised by high triglyceride levels and decreased HDL-C, which can be observed many years before the onset of clinically-relevant hyperglycaemia [32, 33]. The relatively normal total and LDL-C levels can hide an atherogenic lipid profile, with increased small dense LDL typical of dyslipidaemia in diabetes [34, 35].

Although it has been previously demonstrated that 1hPG is associated with inflammatory markers (e.g.: hsCRP) [36] this was not found in our study, probably because our sample is more homogeneous and also because a large number of patients had a chronic level of inflammation.

This study has limitations that should be mentioned. The included patients came from a tertiary hospital, and they were referenced for BS, which can skew the results as it is a very specific population, moreover, these results may not be generalizable to populations of different traits. Therefore, further studies including a more diversified sample of patients should be taken to corroborate our findings. Furthermore, the impact of the found changes in each method on the progression to T2DM after BS has not been evaluated, which limit the conclusions concerning the accuracy of the methods in predicting T2DM progression. Moreover, data on carotid echocardiography, ABI, vascular endothelial function that would help on identifying potential cardiometabolic risk factors, were not available for this study population, and should also be considered a potential study limitation. Additionally, although most of the recuited patients were female (86.9%) this percentage is in agreement with the the sex distribution of patients who attended bariatric appointments.

This study showed that 1hPG could be an alternative for the diagnosis of prediabetes in morbidly obese patients. Considering the paucity of research regarding the agreement between 1 and 2hPG in people with morbid obesity, this study provided relevant insights on this regard. Also, this study was strengthened by the large number of subjects included.

## Conclusions

A fair agreement between 1 and 2hPG was found. 1hPG may be useful for evaluating β-cell dysfunction and IR in morbidly obese patients without diagnosed diabetes. Abnormalities in 1hPG could represent a more severe metabolic perturbation, which is characterised by higher glycaemic, lower insulin sensitivity, and a markedly reduced β-cell function.

## Data Availability

The datasets used and/or analysed during the current study are available from the corresponding author on reasonable request.

## Abbreviations

1Hpg: 1-Hour plasma glucose concentration
2hPG: 2-Hour plasma glucose concentration
BMI: Body mass index
BS: Bariatric surgery
CVD: Cardiovascular disease
DBP: Diastolic blood pressure
FPG: Fasting plasma glucose
HbA1c: Glycated haemoglobin
HC: Hip circumference
HDL-c: High-density lipoprotein cholesterol
HOMA-β: Homeostatic model assessment for cell-β function
HOMA-IR: Homeostatic model assessment for insulin resistance
hsCRP: High sensitivity C-reactive protein
IQR: Interquartile range
LABG: Laparoscopic adjustable gastric band
LDL-c: Low-density lipoprotein cholesterol
LSG: Laparoscopic sleeve gastrectomy
NGT: Normal glucose tolerance
OGTT: Oral glucose tolerance test
PG: Post-load glucose
RYGB: Roux-en-Y gastric bypass
SBP: Systolic blood pressure
SD: Standard deviation
T2DM: Type 2 diabetes *mellitus*
WC: Waist circumference
